# RETRACTION: “The Proliferation Enhancing Effects of Salidroside on Schwann Cells In Vitro”

**DOI:** 10.1155/ecam/9857212

**Published:** 2025-04-24

**Authors:** Evidence-Based Complementary and Alternative Medicine

RETRACTION: H. Liu, P. Lv, H. Wu, K. Zhang, F. Xu, L. Zheng, and J. Zhao, “The Proliferation Enhancing Effects of Salidroside on Schwann Cells In Vitro,” *Evidence-Based Complementary and Alternative Medicine*, vol. 2017 (2017). https://doi.org/10.1155/2017/4673289.

The above article, published online on 7 June 2017 in Wiley Online Library (https://wileyonlinelibrary.com), has been retracted by John Wiley & Sons Ltd.

The retraction has been agreed following an investigation into concerns originally raised by *Actinopolyspora biskrensis* on PubPeer [1], which identified concerns in Figure 4(a).

More specifically, similarity has been identified between the 6-day control panel and the 6-day S-3 panel. The figures are mostly identical; however, there are some key inconsistencies.

As a result of this investigation, the conclusions of the article are considered unreliable.

Li Zheng and Jinmin Zhao disagree with this retraction. The other authors were informed of this decision but did not provide a response.
